# Supplementary figures and images for: Within-host adaptive speciation of commensal yoyo clams leads to ecological exclusion, not co-existence
Source: PeerJ. 2024 Aug 5;12:e17753. doi: 10.7717/peerj.17753 (PMC11308998; doi:10.7717/peerj.17753)

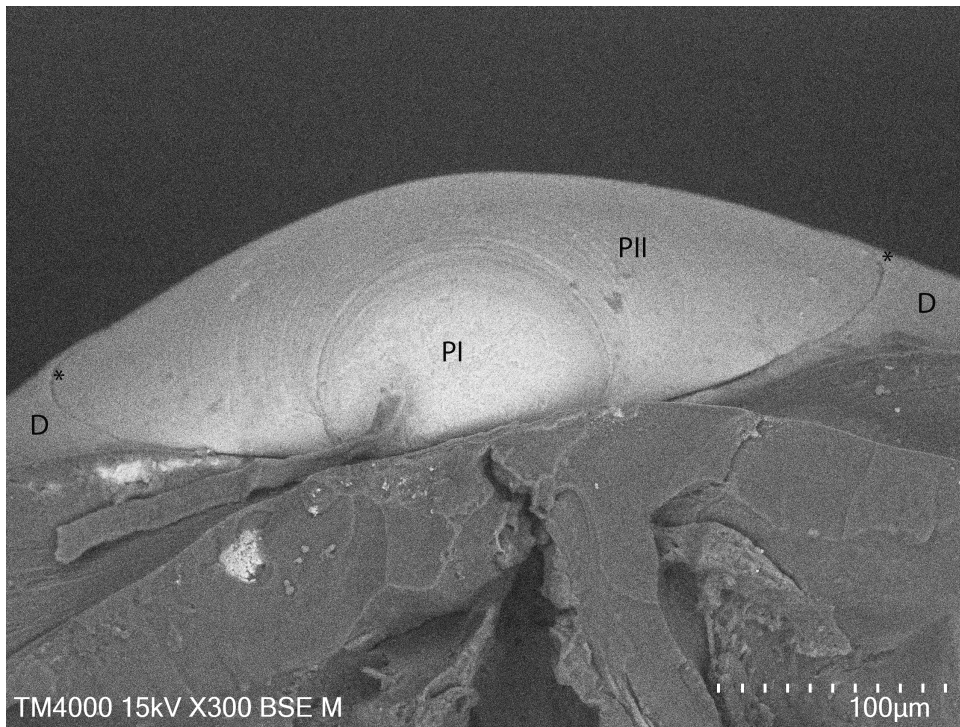

Supplement: Supplemental Information 1 — PI indicates the D-shaped Prodissoconch I (126 µm in length); PII shows the much larger Prodissoconch II (370 µm in length) characterized by commarginal growth lines; D indicates the post-larval, juvenile Dissoconch shell formed after metamorphosis, and the two *s locate the metamorphic line separating the larval and juvenile shell portions. The PI/PII length ratio of this specimen was 126/370 µm, or 34% [indicating planktotrophic larval development Ockelmann, 1965] and it falls between the respective values Mikkelsen & Bieler (1992) recorded for Divariscintilla octotentaculata (115/360 µm or 32%) and for D. luteocrinita (145/390 µm or 37%), two closely related species with confirmed planktotrophic larval development. “P.” squillina’s larval shell morphology is therefore entirely consistent with it undergoing planktotrophic larval development and dispersal. [file peerj-12-17753-s001.pdf]

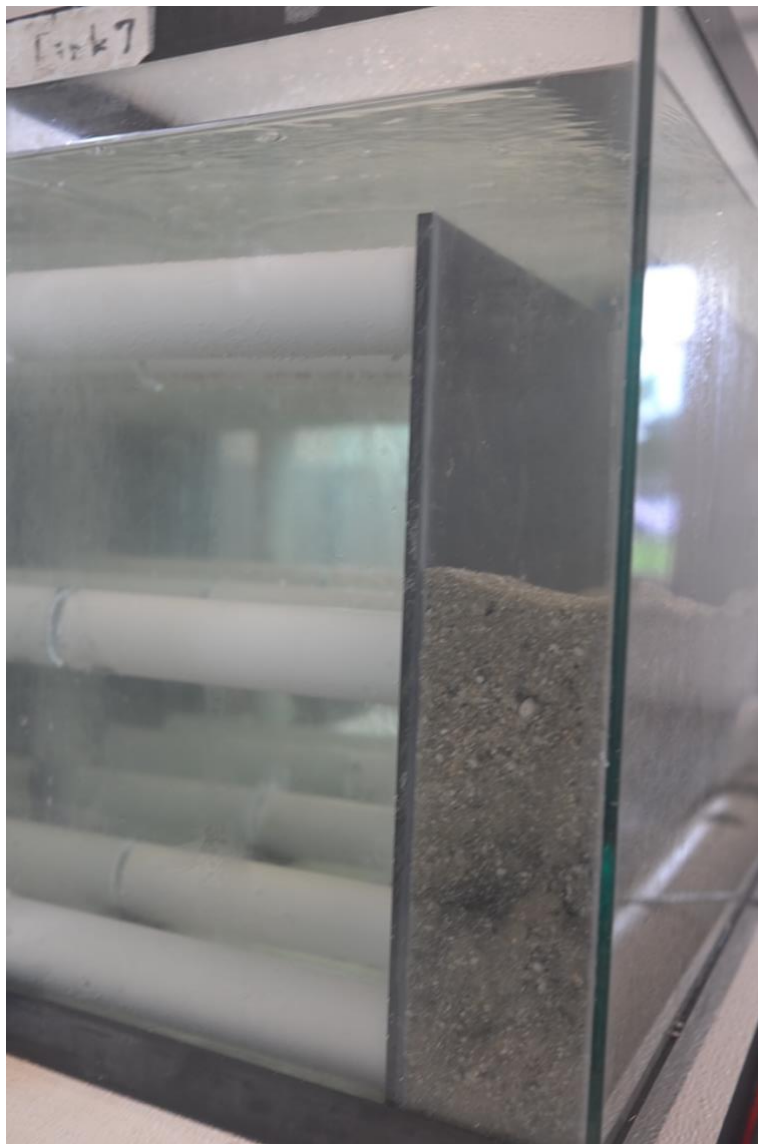

Supplement: Figure S2 — Lateral view of an aquarium tank showing the white PVC support structures holding up a thin wedge of sediment that housed an artificial host burrow. [file peerj-12-17753-s002.pdf]
